# Editorial: Monitoring and promoting physical exercise and physical performance in esports players

**DOI:** 10.3389/fpubh.2026.1843146

**Published:** 2026-05-18

**Authors:** Bojan Masanovic, Selcuk Akpinar, Dario Novak, Juel Jarani, Mucahit Fisne

**Affiliations:** 1 Faculty for Sport and Physical Education, University of Montenegro, Niksic, Montenegro; 2 Western Balkan Sport Innovation Lab, Podgorica, Montenegro; 3 Physical Education and Sports Department, Nevşehir Haci Bektaş Veli University, Nevşehir, Türkiye; 4 Department of General and Applied Kinesiology, University of Zagreb, Zagreb, Croatia; 5 Faculty of Movement Sciences, Sports University of Tirana, Tirana, Albania; 6 Department of Sport Management, Faculty of Sports Sciences, Sivas Cumhuriyet University, Sivas, Türkiye

**Keywords:** esports athletes, monitoring systems, physical activity, physical fitness, sports performance

## 1 Introduction

The rapid growth and increasing professionalization of esports have drawn greater attention to the health of esports players, which is becoming an important public health concern. Prolonged participation in esports may increase the risk of several health problems, including obesity, musculoskeletal disorders, and chronic diseases (1, 2). These risks are largely linked to prolonged sedentary behavior and irregular sleep patterns commonly reported among esports players (3). Additional concerns include eye strain as well as mental health issues, including gaming disorder (4, 5), which has been officially recognized by the World Health Organization (6). Competitive gamers frequently spend long periods in front of screens such as mobile devices, computers, and gaming consoles, with reports indicating between 10 and 12 h of daily inactivity (7). Growing evidence suggests that such sedentary patterns are associated with adverse health outcomes (8). At the same time, research indicates that regular physical exercise may provide important benefits for both health and physical performance in esports players (9). Moreover, several studies suggest that exercise could represent an effective intervention strategy for improving esports performance through physiological as well as cognitive benefits (10).

Given these considerations, there is a clear need for initiatives aimed at raising awareness and educating gamers about the importance of balanced lifestyle habits, including regular physical activity and adequate mental health support. In this context, this Research Topic was created to provide an opportunity to publish high-quality research focused on monitoring and promoting physical exercise and physical performance in the esports population.

## 2 Contribution to the field

The purpose of this Research Topic is to build a collection of new knowledge related to the effects of women's physical activity across different life stages and to provide practical and theoretical insights that can contribute to science, policy, and practice. This Research Topic aims to consolidate and expand emerging knowledge on the role of physical exercise and physical performance monitoring in esports players and to highlight potential strategies for promoting healthier lifestyle behaviors within this rapidly growing population. The 13 studies published within this Research Topic—comprising one Study Protocol, one Correction, eight Original Research papers (including five cross-sectional studies, one randomized controlled trial, one case study, and one experimental crossover study), and three Review papers (including one content analysis, one scoping review, and one narrative review)—offer readers a valuable opportunity to advance their understanding of this evolving research field. The original research articles included a total of 1,048 participants from eight countries (Türkiye, United States, Austria, and China), ranging in age from 14 to 62 years. In addition, the review studies synthesized evidence from more than 21 primary studies involving over 2,626 participants across more than five countries (Serbia, Australia, England, United States, and Lebanon), representing diverse populations including children, adolescents, adults, older adults, and individuals with disabilities. It should be noted that the actual number of participants and included studies in the three review papers may be higher than reported. In several cases, the authors of these studies did not clearly specify the number of participants, the number of included studies, or the age ranges of the analyzed samples. Such omissions represent limitations that could reflect oversights during the review process and highlight the need for greater methodological transparency and more rigorous reporting standards in future publications.

The studies included in this Research Topic span a wide spectrum of methodological approaches, themes, and age groups, which allowed for several possible ways of classification and interpretation. Ultimately, we structured them into three thematic categories: (1) monitoring and observational studies, describing behavioral patterns and health indicators among esports players; (2) studies examining the effects of physical activity, gaming exposure, and gaming technologies on health and performance outcomes; and (3) studies addressing methodological, technological, and organizational capacities for the implementation and advancement of physical activity programs.

The first group comprised seven studies addressing the monitoring of physical activity, lifestyle behaviors, and health indicators in esports players (one Study Protocol, one Correction, three cross-sectional studies, one content analysis, and one case study). Their findings highlight: the lack of robust methodological approaches to assess physical and health parameters in esports players and emphasize the importance of establishing comprehensive monitoring systems (Long et al.; Long et al.; Zhou et al.); low levels of physical activity, high sedentary behavior, a large number of hours per day spent in a seated position, poor sleep quality, and an increased risk of negative physical health outcomes among esports players (Kusan et al.; Scoggins et al.; Başoglu; Pang et al.).

The second group consisted of four studies examining the effects of physical activity, gaming exposure, and gaming technologies on health and performance outcomes (two cross-sectional studies, one Randomized Controlled Trial, and one experimental crossover study). Their findings suggest: physical activity improves physical performance and wellbeing and reduces social anxiety and sleep problems, although it does not significantly enhance short-term esports performance parameters (Wachholz et al.; Solmaz et al.); frequent digital game playing is associated with faster reaction time but also with weaker physical parameters in the non-dominant shoulder (Güney et al.); virtual reality games produce significantly higher heart rate and physiological load compared to mobile games, suggesting that they may represent a more effective approach for promoting physical activity (Biçer et al.).

The third group included two review studies (one scoping and one narrative review), which were the most difficult to classify, as they focus on the broader infrastructure and tools necessary for advancing research and practice in this field. Their findings indicate: healthcare managers must possess a range of competencies for evidence-based decision-making, including professional and technical knowledge, leadership, communication skills, critical and analytical thinking, and research skills (Sanaeifar et al.); artificial intelligence has considerable potential to support exercise and health programs through the promotion of physical activity, user monitoring, movement analysis, data collection, prediction of health outcomes, and guidance of training processes across diverse populations (Canzone et al.).

## 3 Conclusion

This Research Topic, comprising 13 contributions, reinforces the growing recognition that the health and physical performance of esports players require systematic scientific attention (11). As esports continues to expand globally, understanding how lifestyle behaviors—particularly physical activity, sedentary patterns, and sleep habits—affect the wellbeing and performance of esports players becomes increasingly important. The studies included in this Research Topic help address important knowledge gaps by providing empirical evidence, methodological insights, and practical perspectives relevant to monitoring and promoting physical exercise in esports populations.

Importantly, this Research Topic offers several practical insights that may support the development of healthier esports environments. These include proposals for improved monitoring systems for assessing lifestyle behaviors and health indicators, evidence highlighting the prevalence of sedentary behavior and insufficient physical activity among esports players, and findings demonstrating the potential benefits of physical exercise programs and technology-supported approaches such as virtual reality and artificial intelligence for promoting physical activity and monitoring health outcomes.

Taken together, these findings suggest that promoting regular physical exercise and implementing structured monitoring systems may represent important strategies for mitigating health risks associated with prolonged gaming while also supporting physical and cognitive aspects of esports performance. We hope that the insights presented in this Research Topic will stimulate further interdisciplinary research and contribute to the development of evidence-based strategies for integrating physical activity promotion within the evolving esports ecosystem.
